# Saccharoquinoline, a Cytotoxic Alkaloidal Meroterpenoid from Marine-Derived Bacterium *Saccharomonospora* sp.

**DOI:** 10.3390/md17020098

**Published:** 2019-02-02

**Authors:** Tu Cam Le, Eun Ju Lee, Jihye Lee, Ahreum Hong, Chae-Yoon Yim, Inho Yang, Hyukjae Choi, Jungwook Chin, Sung Jin Cho, Jaeyoung Ko, Hayoung Hwang, Sang-Jip Nam, William Fenical

**Affiliations:** 1 Department of Chemistry and Nano Science, Ewha Womans University, Seoul 03760, Korea; lecamtu5789@gmail.com (T.C.L.); jl3414@gmail.com (J.L.); yimgenie@gmail.com (C.-Y.Y.); 2 New Drug Development Center, Daegu-Gyeongbuk Medical Innovation Foundation (DGMIF), Daegu 41061, Korea; dmswn2588@naver.com (E.J.L.); jwchin@dgmif.re.kr (J.C.); sjcho@dgmif.re.kr (S.J.C.); 3 Laboratories of Marine New Drugs, REDONE Seoul, Seoul 08594, Korea; 4 Graduate School of Industrial Pharmaceutical Sciences, Ewha Womans University, Seoul 03760, Korea; lyzenne@naver.com; 5 Department of Convergence Study on the Ocean Science and Technology, Korea Maritime and Ocean University, Busan 49112, Korea; ihyang@kmou.ac.kr; 6 College of Pharmacy, Yeungnam University, Gyeongsan, Gyeongsangbukdo 38541, Korea; h5choi@yu.ac.kr; 7 Basic Research & Innovation Division Amorepacific R&D Unit, Yongin 17074, Korea; jaeyoungko@amorepacific.com; 8 Center for Marine Biotechnology and Biomedicine, Scripps Institution of Oceanography, University of California-San Diego, La Jolla, CA 92093-0204, USA

**Keywords:** *Saccharomonospora* sp., meroterpenoid, marine natural product, cytotoxicity

## Abstract

A cytotoxic alkaloidal meroterpenoid, saccharoquinoline (**1**), has been isolated from the fermentation broth of the marine-derived bacterium *Saccharomonospora* sp. CNQ-490. The planar structure of **1** was elucidated by 1D, 2D NMR, and MS spectroscopic data analyzes, while the relative configuration of **1** was defined through the interpretation of NOE spectroscopic data. Saccharoquinoline (**1**) is composed of a drimane-type sesquiterpene unit in combination with an apparent 6,7,8-trihydroxyquinoline-2-carboxylic acid. This combination of biosynthetic pathways was observed for the first time in natural microbial products. Saccharoquinoline (**1**) was found to have cytotoxicity against the HCT-116 cancer cell line by inducing G1 arrest, which leads to cell growth inhibition.

## 1. Introduction

**Figure 1:**
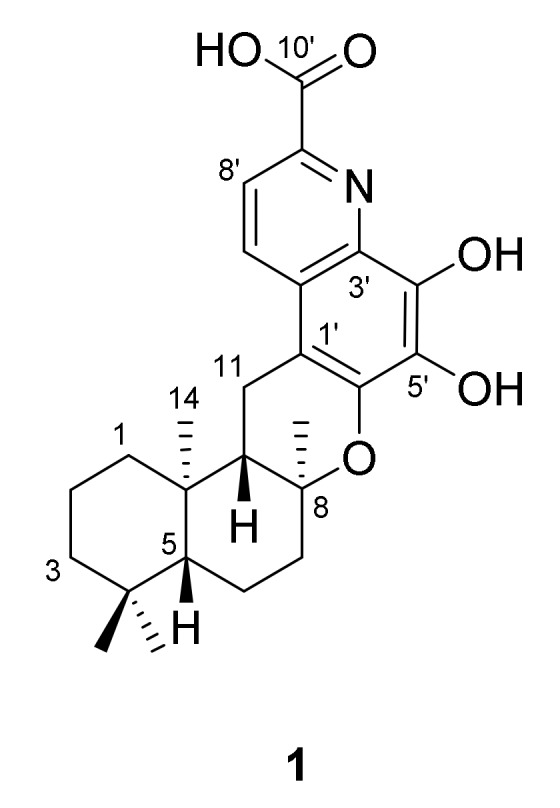
Chemical structure of saccharoquinoline (**1**).

The marine-derived bacterium *Saccharomonospora* sp. CNQ-490 is an actinomycete which, based upon phylogenetic analysis, represents a new operational taxonomic unit within the genus *Saccharomonospora* [1]. Previous chemical investigations of the strain CNQ-490 led to the isolation of a series of alkaloids, namely, the lodopyridones [1,2], which revealed a unique combination of ethanolamine, thiomethyl-substituted 4-pyridone, thiazole, and chloroquinoline moieties within its structure. A further study of this strain also yielded three new α-pyrones—the saccharomonopyrones A–C [3]. Further, a recent genomic study on *Saccharomonospora* sp. CNQ-490 led to the successful heterologous expression of a silent bio-synthetic gene cluster, which lead to the discovery of the new anti-biotic taromycins A and B [4,5]. The observation of 19 apparent biosynthetic gene clusters in this strain also indicated that this actinomycete could be a promising source for additional structurally diverse secondary metabolites [4]. LC-MS analysis on a large-scaled culture crude extract of this strain revealed a peak with UV absorption signals at *λ_max_* 281 and 349 nm, which corresponds to a molecular weight of 425. HPLC-UV-guided purification of the culture extract of the strain yielded an alkaloidal meroterpenoid saccharoquinoline (**1**, Figure 1).

## 2. Results and Discussion

### 2.1. Chemical Structure Elucidation

Saccharoquinoline (**1**) was isolated as a viscous oil by repeated C_18_ silica flash column fractionation and final C_18_ HPLC purifications. The molecular formula of **1** was deduced as C_25_H_31_NO_5_ from the analysis of HRESIMS data in combination with NMR spectroscopic data. A strong IR absorption at 1708 cm^-1^ indicated the presence of carbonyl functionality, which was ultimately assigned as a carboxylic acid. The ^1^H-NMR spectrum of **1** exhibited two doublet ortho-aromatic protons (H-7′ (δ_H_ 8.40, 1H, *J* = 8.6 Hz) and H-8′ (δ_H_ 7.92, 1H, *J* = 8.6 Hz)), and four methyl singlets (H-12 (δ_H_ 0.85), H-13 (δ_H_ 0.90), H-14 (δ_H_ 0.94), and H-15 (δ_H_ 1.18)). The ^13^C NMR and HSQC spectroscopic data also indicated the presence of four methyl singlets (C-12 (δ_C_ 21.5), C-13 (δ_C_ 33.2), C-14 (δ_C_ 14.6), and C-15 (δ_C_ 20.3)), six methylenes (C-1 (δ_C_ 38.5), C-2 (δ_C_ 18.0), C-3 (δ_C_ 41.3), C-6 (δ_C_ 19.3), C-7 (δ_C_ 40.3), and C-11 (δ_C_ 17.9)), two methines (C-5 (δ_C_ 55.3) and C-9 (δ_C_ 51.2)), two aromatic methines (C-7′ (δ_C_ 132.1) and C-8′ (δ_C_ 116.9)), three un-protonated sp^3^ (C-4 (δ_C_ 32.8), C-8 (δ_C_ 78.1), and C-10 (δ_C_ 36.6)), and eight un-protonated sp^2^ carbons (C-1′ (δ_C_ 104.9), C-2′ (δ_C_ 122.8), C-3′ (δ_C_ 133.6), C-4′ (δ_C_ 135.0), C-5′ (δ_C_ 136.9), C-6′ (δ_C_ 145.8), C-9′ (δ_C_ 140.8), and C-10′ (δ_C_ 165.6)) (Table 1).

Further interpretation of the 2D spectroscopic data allowed us to assign the structure of **1**. First, the drimane-type sesquiterpene unit was secured from the analysis of the COSY and HMBC spectroscopic data. COSY correlations (H-1 (δ_H_ 0.99, 1.83)/H-2 (δ_H_ 1.44, 1.66)/H-3 (δ_H_ 1.18, 1.39), H-5 (δ_H_ 1.08)/H-6 (δ_H_ 1.42, 1.74)/H-7 (δ_H_ 1.73, 2.15), and H-9 (δ_H_ 1.67)/H-11 (δ_H_ 2.69, 2.91)), the HMBC correlations from gem dimethyls H_3_-12 and H_3_-13 to C-3, C-4, and C-5, a methyl singlet H_3_-14 to C-1, C-5, C-9, and C-10, and from a methyl singlet H_3_-15 to C-7, C-8, and C-9 permitted the establishment of a drimane moiety.

**Figure 2:**
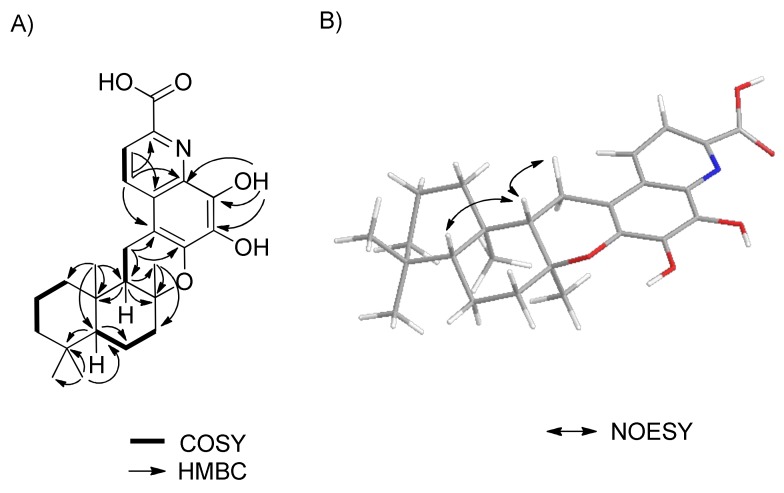
(**A**) COSY and key HMBC correlations. (**B**) Key NOESY correlations of saccharoquinoloine (**1**).

Second, the quinoline moiety could also be constructed by the interpretation of the 1D and 2D NMR spectroscopic data. The COSY spectrum confirmed two ortho-coupled aromatic protons, H-7′ and H-8′. HMBC correlations were observed from H-8′ to C-2′, and from H-7′ to C-1′, C-3′, and C-9′, and from the exchangeable proton 4′-OH to C-3′, C-4′, and C-5′. These observations, coupled with the presence of an aromatic nitrogen, suggested the presence of a quinoline moiety. The typical carbon chemical shifts of C-5′ (δ_C_ 136.9) and C-6′ (δ_C_ 145.8) indicated the attachment of oxygen atoms to C-5′ and C-6′. HMBC NMR correlations from methylene H_2_-11 to C-1′ and C-6′ permitted the connectivity of C-9/C-11/C-1′ and the typical oxygenated carbon chemical shift of C-8 (δ_C_ 78.1) indicated the connectivity of C-8 and C-6′ through an oxygen atom. Even though no relevant HMBC correlations were observed for the carboxylic group of C-10′, this moiety was placed at C-9′, which was based on the observation of ortho-coupled protons for protons H-7′ and H-8′. Quinoline-2-carboxylic acids are common metabolites and their spectroscopic data are well known. Thus, the planar structure of saccharoquinoline (**1**) was determined, as shown in Figure 2A.

The complete relative stereo-structure of **1** was readily derived by the interpretation of NOESY NMR correlations of the drimane skeleton. NOESY correlations between H-9 and H-11β (δ_H_ 2.69), and H-5 and H-9 indicated that those protons (H-5, H-9, and H-11β) were located on the same side of the tricyclic ring system (Figure 2B).

Saccharoquinoline (**1**) is an alkaloidal meroterpenoid composed of a common drimane sesquiterpene and an unreported 6,7,8-trihydroxyquinoline-2-carboxylic acid moiety. Searches of the Antimarin Database showed 237 secondary metabolites containing the quinoline moiety isolated from microorganisms, but there were no reports of compounds having a hybrid structure, as in **1**. The closest chemical structure to **1** is that of thallusin (**2**), which was isolated from the marine bacterium *Flavobacterium* sp. in 2005 [6]. Thallusin (**2**) has a broken ring structure between the C-4′ and C-5′ of **1,** with a further oxidation state that has two carboxylic acids. The initial isolation of the thallusin was followed by several synthetic efforts [7,8]. In 2014, a chiral synthetic route based on enzymatic hydrolysis was developed to correct the originally proposed stereo-structure [9]. 

The structural similarity of these two meroterpenoids implies that they share the same biosynthetic pathway; however, they have an additional oxidative cleavage of the C-4′–C-5′ bond. 

**Figure 3:**
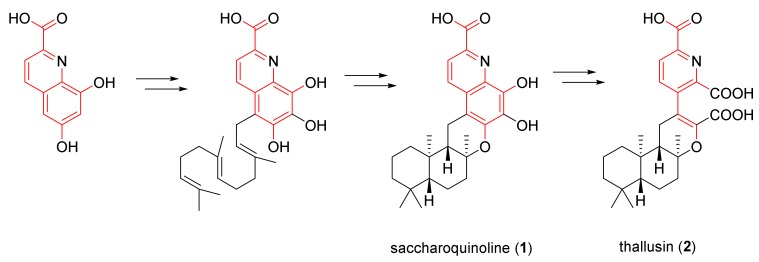
Plausible biosynthetic pathway for saccharoquinoline (**1**) and thallusin (**2**).

It is not clear how these two metabolites are related. On the basis of feasible chemical conversions, the vicinal aromatic diol of **1** can be oxidized to the ring open dicarboxylic acid, in a fashion that is analogous to the oxidation of catechol to muconic acid [10]. Saccharoquinoline (**1**) and thallusin (**2**) show rare quinoline starting at the terpenoid pathway (Figure 3). A quinoline moiety from lodopyridones, which was isolated from the same strain, also supports this hypothesis [1,2].

### 2.2. Bioactivities

**Figure 4:**
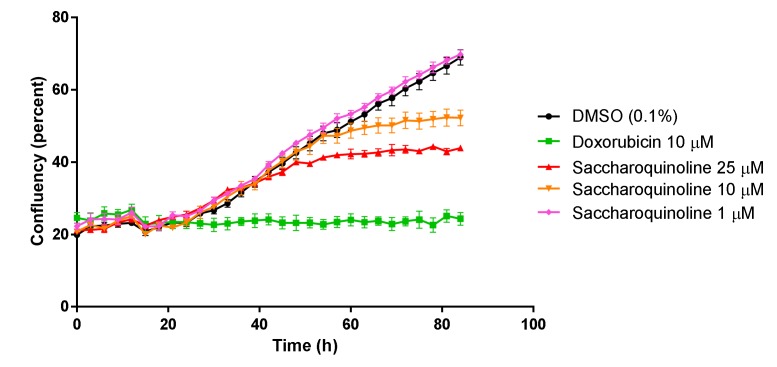
Colon cancer cell (HCT116) growth in saccharoquinoline (**1**)-treated and vehicle-treated (DMSO, 0.1%) cells measured using live cell imaging. Doxorubicin treated cells were used as a control for cytotoxicity.

**Figure 5:**
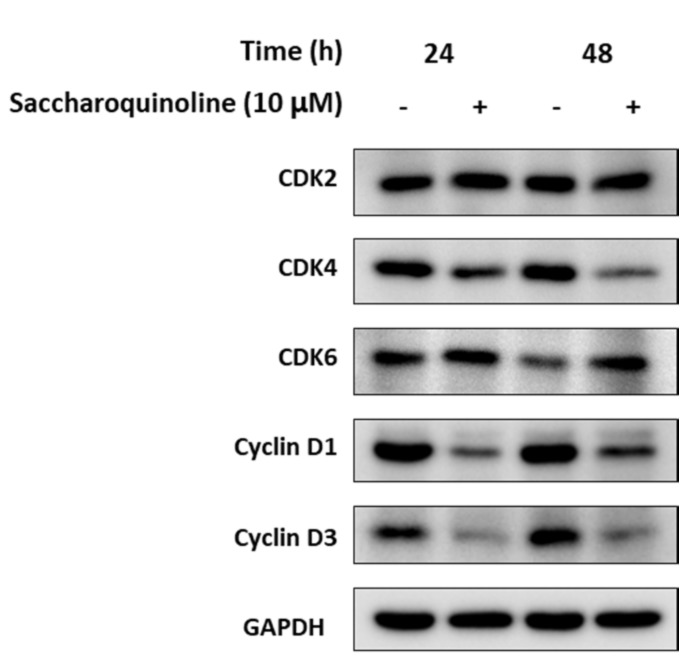
Downregulation of cell cycle checkpoint proteins involved in the G1 phase transition to the S phase. HCT116 cells were treated with **1** (10 μM, +) for 24 hours or 48 hours, and with vehicle (DMSO, 0.1%). The expressions of CDK2, CDK4, CDK6, cyclin D1, and cyclin D3 were illustrated by Western blot analysis. GAPDH was used as the internal control.

Next, we tested saccharoquinoline (**1**) for its cytotoxic effect in diverse tumor-originating cancer cell lines such as H1299, A549, HCT116, PC-3, HepG2, and in the BJ normal cell line [11]. At a concentration of 10 μM, **1** induced cell growth inhibition of approximately 50% in H1299 and HCT116. The treatment at the highest concentration of 25 μM showed strong inhibition in the H1299, HCT116, and PC-3 cell lines, but also induced cell mortality of approximately 43% in the BJ cells (Table 2). Among the tested cancer cell lines, we chose the HCT116 colon cancer cells, which showed a high cytotoxic response, and confirmed the growth inhibition of **1** by using live cell growth measurements. As shown in Figure 4, **1** inhibited HCT-116 in a dose-dependent manner. Cell cycle regulation, and particularly G1 arrest, is one of the origins of cytotoxicity. Therefore, we compared the expressions of G1 check proteins such as CDKs and cyclins in the **1**-treated and control cells. Figure 5 depicts the strong inhibition of cyclins D1 and D3, which play an important role in the transition from the G1 phase to the S phase. These data showed that saccharoquinoline (**1**) induced G1 arrest by the downregulation of cyclins D1 and D3 in colon cancer cells, resulting in cell growth inhibition [12].

## 3. Materials and Methods

### 3.1. General Experimental Procedures

An optical rotation was measured on an Autopol III (Rudolph Research Analytical, Hackettstown, NJ, USA) polarimeter with a 5-cm cell, and the UV spectra were recorded on a Scinco UVS-2100 spectrophotometer (Sinco, Daejeon, Korea). IR spectra were taken on a Perkin-Elmer 1600 FT-IR spectrometer (Waltham, MA, USA). NMR spectra were recorded on a Varian Inova NMR spectrometer (Varian Inc., Palo Alto, CA, USA; 300 and 75 MHz for ^1^H and ^13^C NMR, respectively) using the signals of the residual solvent as internal references; δ_H_ 2.50 and δ_C_ 39.5 ppm for dimethyl sulfoxide-*d*_6_ (DMSO-*d*_6_). 2D NMR spectra were recorded on a Varian Inova 500 MHz NMR spectrometer (Varian Inc., Palo Alto, CA, USA). High-resolution ESIMS spectra were obtained using a JEOL JMS-AX505WA mass spectrometer (JEOL Ltd. Tokyo, Japan). Low-resolution LC-MS data were acquired using an Agilent Technologies 6120 quadrupole LC/MS system (Agilent Technologies, Santa Clara, CA, USA) with a reversed-phase column (Phenomenex Luna C18 (2) 100 Å, 50 mm × 4.6 mm, 5 μm) at a flow rate of 1.0 mL/min. Open column chromatography was performed on C_18_ silica (40–63 μm, ZEO prep 90) with a gradient solvent of water (H_2_O) and methanol (MeOH).

### 3.2. Strain Isolation and Fermentation

Strain CNQ-490 was isolated from a sediment sample collected at a depth of 45 m, 2 km west of the Scripps pier in La Jolla, CA. The 16S rDNA sequence of this strain showed the closest identification with the genus *Saccharomonospora* (GenBank accession number EU214929). A 40-L culture of the strain CNQ-490 was performed at RT using 2.5-L Ultra Yield Flasks (Thomson Scientific, Oceanside, CA, USA), each containing 1 L of the medium (10 g/L of soluble starch, 2 g/L of yeast, 4 g/L of peptone, 10 g/L of CaCO3, 20 g/L of KBr, 8 g/L of Fe_2_(SO_4_)_3_·4H_2_O dissolved in 1000 mL of artificial seawater) at 25 °C with shaking at 150 rpm. After 7 days of culture, the whole culture broth was extracted with ethyl acetate (EtOAc), and the solvent was removed in vacuo to yield 2.2 g of EtOAc extract.

### 3.3. Extraction and Purification

The organic extract was first fractionated by reversed-phase C_18_ silica flash chromatography using step-gradient elution (80% of H_2_O in MeOH to 100% MeOH, and 500 mL each, but the last solvent, which was 1000 mL, had two fractions) to yield 10 fractions. Fraction 7, eluted with 80% of MeOH in water, contained saccharoquinoline (**1**). This fraction (112 mg) was purified by reversed-phase HPLC under isocratic conditions with 60% of acetonitrile in H_2_O to yield 2.5 mg of **1** (monitored with a UV detector at 281 nm and a retention time of 43.5 minutes).

*Saccharoquinoline* (**1**): Yellowish oil, [α]_D_^25^ + 55 (c 0.002, MeOH); UV (MeOH) *λ_max_* (log ε) 200 (2.15), 281 (2.09), and 349 (1.24) nm; IR (KBr) ν_max_ 3422, 2927, 1708, 1624, and 1452 cm^-1^; ^1^H and ^13^C NMR data, Table 1 and Supplementary Materials; HRESIMS *m/z* 426.2289 [M + H]^+^ (calcd for C_25_H_32_NO_5_, 426.2275).

### 3.4. Cytotoxicity Test

Cancer cell lines, obtained from ATCC (VA, USA), were cultured in a basal culture media containing 10% FBS (GE Healthcare Life Sciences, Pittsburgh, PA, USA) and 1% penicillin/streptomycin (GE Healthcare Life Sciences, Pittsburgh, PA, USA). The basal culture media used was MaCoys’ 5A (Life Technologies, Carlsbad, MA, USA) for HCT116 (ATCC CCL-247), RPMI (Life Technologies, Carlsbad, MA, USA) for H1299 (ATCC CRL-5803), and DMEM (Life Technologies, Carlsbad, MA, USA) for A549 (ATCC CCL-185), PC-3 (ATCC CRL-1435), and HepG2 (ATCC HB-8065). The normal human fibroblast cell line, BJ (ATCC CRL-2522), was cultured in DMEM supplemented with 10% FBS and 1% penicillin/streptomycin. For the cytotoxicity test, cells with a total of 2.5 × 10^3^ per well of the 96 well culture plate were seeded, and on the next day, saccharoquinoline (**1**) was treated as 1, 10, and 25 μM in a basal media containing 1% FBS with a final concentration of 0.1% DMSO. Three days (72 h) after treatment, 100 μL Cell Titer Glo reagent (Promega, Madison, WI, USA) was added to each well and luminescence activity was measured using a multifunctional microplate reader (Tecan, Männedorf, Switzerland). Cell viability was analyzed as percent (%) viability compared with the DMSO (0.1%)-treated control. Doxorubicin was used as a positive control. For HCT116 cell growth analysis in the live state, cells were seeded to be the number of 2.5 × 10^3^ in each well of a 96-well plate, and cell growth, with or without treatment of **1**, was monitored for 4 days. Cell growth was measured by the percent (%) of cell confluence in each well of a 96-well plate using the IncuCyte ZOOM program (Sartorius, Göttingen, Germany).

### 3.5. Western Blot

Saccharoquinoline (**1**, 10 μM, with 0.1% DMSO) or vehicle (DMSO, 0.1%) was added to HCT116 cells cultured in a basal culture media supplemented with 1% FBS. Cells were harvested 24 h and 48 h after treatment and were lysed with RIPA (radioimmunoprecipitation assay) buffer (Roche, Basel, Switzerland) containing protease and phosphatase inhibitors (Roche). Cell lysates were kept on ice for 30 min and centrifuged at 15,000 rpm for 20 min. Then, supernatants were transferred into new tubes and the protein concentrations were measured using a BCA protein assay kit (Thermo Fisher Scientific, Waltham, MA, USA). Next, the protein extracts (30 μg) were electrophoresed and transferred to a PVDF membrane (EMD Millipore, Burlington, MA, USA). Then, the membrane was incubated with primary antibodies against CDK2, CDK4, CDK6, cyclin D1, cyclin D3, and GAPDH, purchased from Cell Signaling Technology (Danvers, MA, USA), at 4 °C overnight. The primary antibodies were detected by incubation with a horseradish peroxidase-conjugated secondary antibody (Bethyl Laboratories, Montgomery, TX, USA) at 25 °C for 1 h. Bindings of antibodies were visualized by an ImageQuant LAS4000 imager (GE Healthcare Life Sciences) after incubation with an ECL substrate (Bio-Rad, Hercules, CA, USA).

## 4. Conclusions

In conclusion, a cytotoxic alkaloidal meroterpenoid, saccharoquinoline (**1**), was isolated from *Saccharomonospora* sp. CNQ-490 as the first candidate for a hybrid structure between a drimane sesquiterpene and the rare 6,7,8-trihydroxyquinoline-2-carboxylic acid. The successful discovery of **1** along with the diversity of previously reported secondary metabolites from this *Saccharomonospora* strain, coupled with the genomic biosynthetic gene cluster information from previous studies, emphasizes the significant biosynthetic potential of the strain.

## Figures and Tables

**Table 1:** NMR spectroscopic data for saccharoquinoline (**1**) in DMSO-*d*_6_, δ in ppm ^a^.

No.	1			
	*δ*_C_, mult. ^b^	*δ*_H_ (*J* in Hz)	COSY	HMBC
1	38.5, CH_2_	0.99, brd; 1.83, d (14.3)	2	
2	18.0, CH_2_	1.44, m ^c^; 1.66, m ^c^	1, 3	
3	41.3, CH_2_	1.18 ^c^; 1.39 ^c^	2	
4	32.8, C			
5	55.3, CH	1.08 ^c^		4, 6, 10, 12, 14
6	19.3, CH_2_	1.42, m ^c^; 1.74, m^c^	7	
7	40.3, CH_2_	1.73 ^c^; 2.15, dt (12.3, 3.6)	6	
8	78.1, C			
9	51.2, CH	1.67 ^c^	11	8, 10, 11, 14, 15
10	36.6, C			
11	17.9, CH_2_	2.69, dd (16.8, 5.2); 2.91, dd (16.8, 13.1)	9	8, 9, 1′, 6′
12	21.5, CH_3_	0.85, s		3, 4, 5, 13
13	33.2, CH_3_	0.90, s		3, 4, 5, 12
14	14.6, CH_3_	0.94, s		1, 5, 9, 10
15	20.3, CH_3_	1.18, s		7, 8, 9
1′	104.9, C			
2′	122.8, C			
3′	133.6, C			
4′	135.0, C			
5′	136.9, C			
6′	145.8, C			
7′	132.1, CH	8.40, d (8.6)	8′	1′, 3′, 9′
8′	116.9, CH	7.92, d (8.6)	7′	2′
9′	140.8, C			
10′	165.6, C			
4′-OH		9.54, s		3′, 4′, 5′

^a^ 300 MHz for ^1^H NMR and 75 MHz for ^13^C NMR. ^b^ Numbers of attached protons were determined by analysis of 2D NMR spectroscopic data (500 MHz). ^c^ Overlapping signals.

**Table 2:** Viability assay in several types of cancer cells and normal cells after treatment with saccharoquinoline (**1**) for 72 h.

		Cancer					Normal
	Concentration (μM)	Lung		Colon	Prostate	Liver	Skin
		H1299	A549	HCT116	PC-3	HepG2	BJ
DMSO (0.1%)		100	100	100	100	100	100
Doxorubicin	10	6	5	11	24	7	16
**1**	1	87	103	89	106	98	87
	10	45	100	39	88	82	80
	25	29	81	25	46	71	57

## Supplementary Materials

The following are available online at http://www.mdpi.com/1660-3397/17/2/98/s1, Figure S1: ^1^H NMR spectrum (300 MHz) of saccharoquinoline (**1**) in DMSO-*d*_6_; Figure S2: ^13^C NMR spectrum (75 MHz) of saccharoquinoline (**1**) in DMSO-*d*_6_; Figure S3: gCOSY spectrum (500 MHz) of saccharoquinoline (**1**) in DMSO-*d*_6_; Figure S4: gHSQC spectrum (500 MHz) of saccharoquinoline (**1**) in DMSO-*d*_6_; Figure S5: gHMBC spectrum (500 MHz) saccharoquinoline (**1**) in DMSO-*d*_6_; Figure S6: gNOESY spectrum (500 MHz) of saccharoquinoline (**1**) in DMSO-*d*_6_.
