# Supplementary material for: Saccharoquinoline, a Cytotoxic Alkaloidal Meroterpenoid from Marine-Derived Bacterium Saccharomonospora sp
Source: Mar Drugs. 2019 Feb 2;17(2):98. doi: 10.3390/md17020098 (PMC6410326; doi:10.3390/md17020098)

Supporting Information for

Saccharoquinoline, a Cytotoxic Alkaloidal Meroterpenoid from Marine-Derived Bacterium *Saccharomonospora* sp.

Tu Cam Le [1,†], Eun Ju Lee [2,†], Jihye Lee [1,†], Ahreum Hong [3], Chae-Yoon Yim [1], Inho Yang [4], Hyukjae Cho [5], Jungwook Chin [2], Sung Jin Cho [2], Jaeyoung Ko [6], Hayoung Hwang [2,*], Sang-Jip Nam [1,*] and William Fenical [7,*]

[1] Department of Chemistry and Nano Science, Ewha Womans University, Seoul 03760, Korea; e-mail@e-mail.com; lecamtu5789@gmail.com (T.L.), jl3414@gmail.com (J.L.), yimgenie@gmail.com (C.Y.)
[2] New Drug Development Center, Daegu-Gyeongbuk Medical Innovation Foundation (DGMIF), Daegu 41061, Korea; dmswn2588@naver.com (E.J.L.), jwchin@dgmif.re.kr (J.C.), sjcho@dgmif.re.kr (S.J.C.),
[3] Graduate School of Industrial Pharmaceutical Sciences, Ewha Womans University, Seoul 03760, Korea; lyzenne@naver.com
[4] Department of Convergence Study on the Ocean Science and Technology, Korea Maritime and Ocean University, Busan 49112, Korea; ihyang@kmou.ac.kr
[5] College of Pharmacy, Yeungnam University, Gyeongsan, Gyeongsangbukdo 38541, Korea; h5choi@yu.ac.kr
[6] Basic Research & Innovation Division Amorepacific R&D Unit, Yongin 17074, Korea; jaeyoungko@amorepacific.com
[7] Center for Marine Biotechnology and Biomedicine, Scripps Institution of Oceanography, University of California-San Diego, La Jolla, CA 92093-0204, USA
[*] Correspondence: hwanghy@dgmif.re.kr (H.H.), sjnam@ewha.ac.kr (S.-J.N.), wfenical@ucsd.edu (W.F.)
[†] These authors contributed equally to this work

Table of Contents

Figure S1. ^{1}H NMR Spectrum (300 MHz) of saccharoquinoline (**1**) in DMSO-d_6…………………........................... S3

Figure S2. ^{13}C NMR Spectrum (75 MHz) of saccharoquinoline (**1**) in DMSO-d_6 ……………………………………. S4

Figure S3. gCOSY Spectrum (500 MHz) of saccharoquinoline (**1**) in DMSO-d_6 ……………………………………. S5

Figure S4. gHSQC Spectrum (500 MHz) of saccharoquinoline (**1**) in DMSO-d_6 ……………………………………. S6

Figure S5. gHMBC Spectrum (500 MHz) saccharoquinoline (**1**) in DMSO-d_6 ………………………………………. S7

Figure S6. gNOESY Spectrum (500 MHz) of saccharoquinoline (**1**) in DMSO-d_6 …………………………………... S8

Figure S1. ^{1}H NMR Spectrum (300 MHz) of saccharoquinoline (**1**) in DMSO-d_6

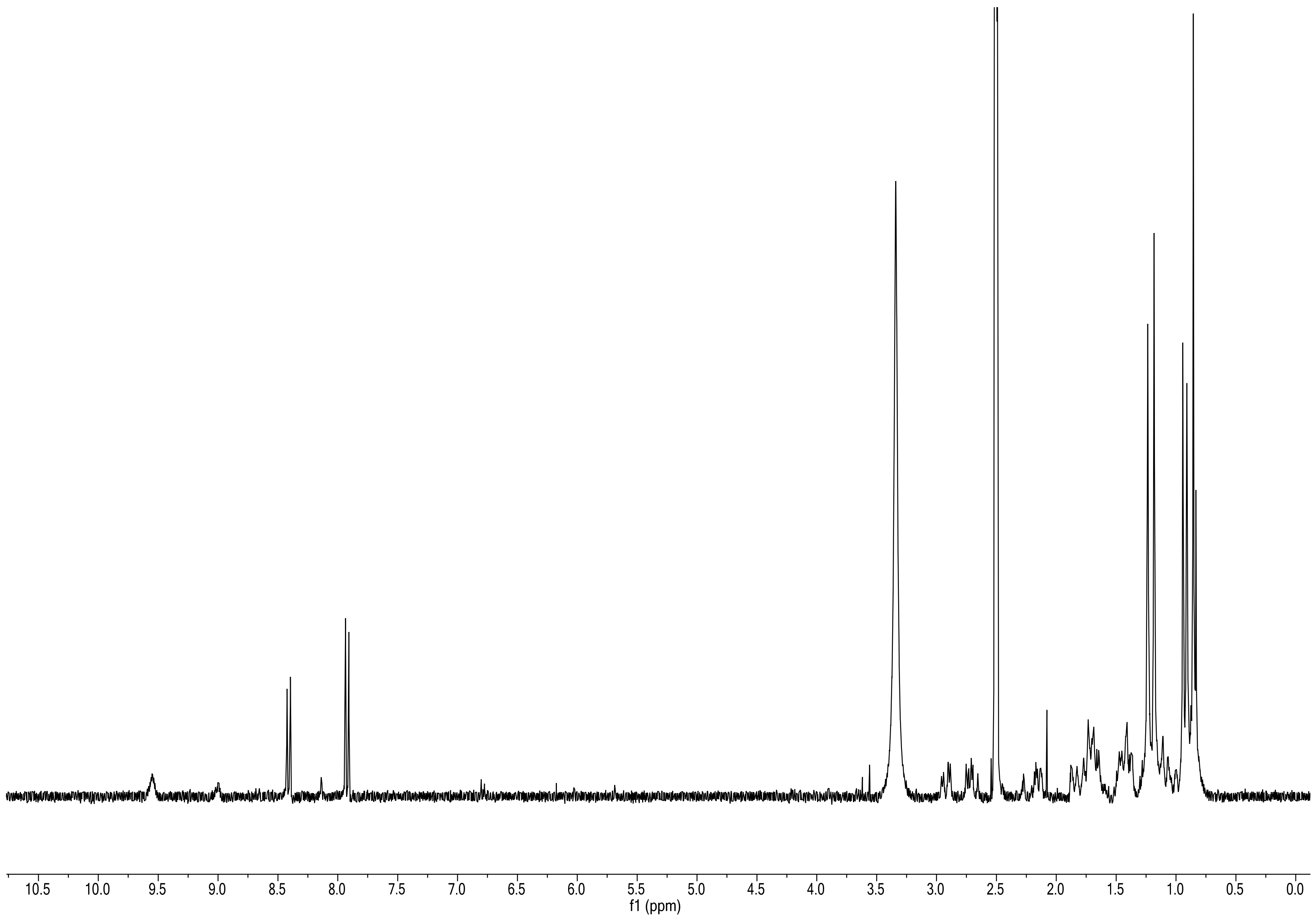

Figure S2. ^{13}C NMR Spectrum (75 MHz) of saccharoquinoline (**1**) in DMSO-d_6

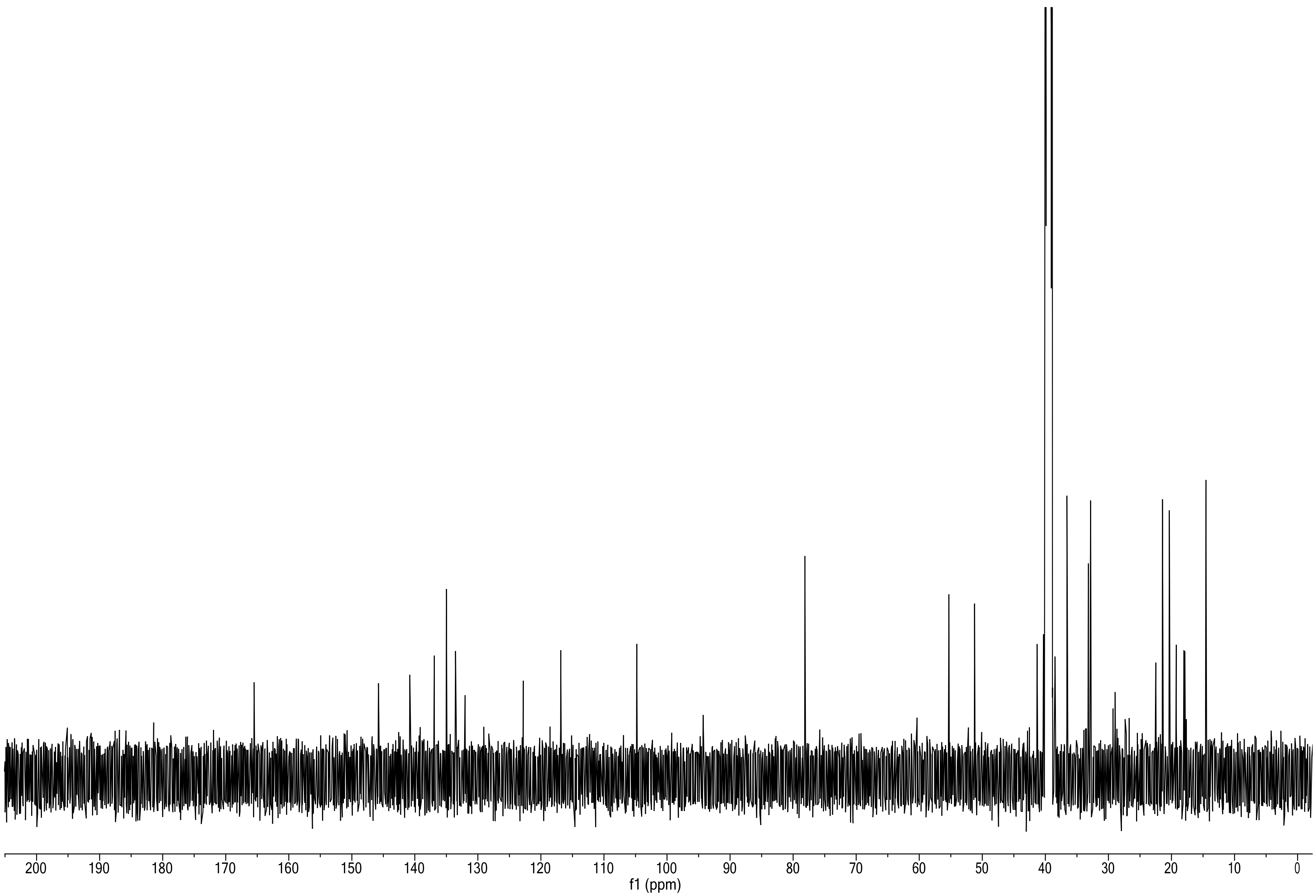

Figure S3. gCOSY Spectra (500 MHz) of saccharoquinoline (**1**) in DMSO-d_6

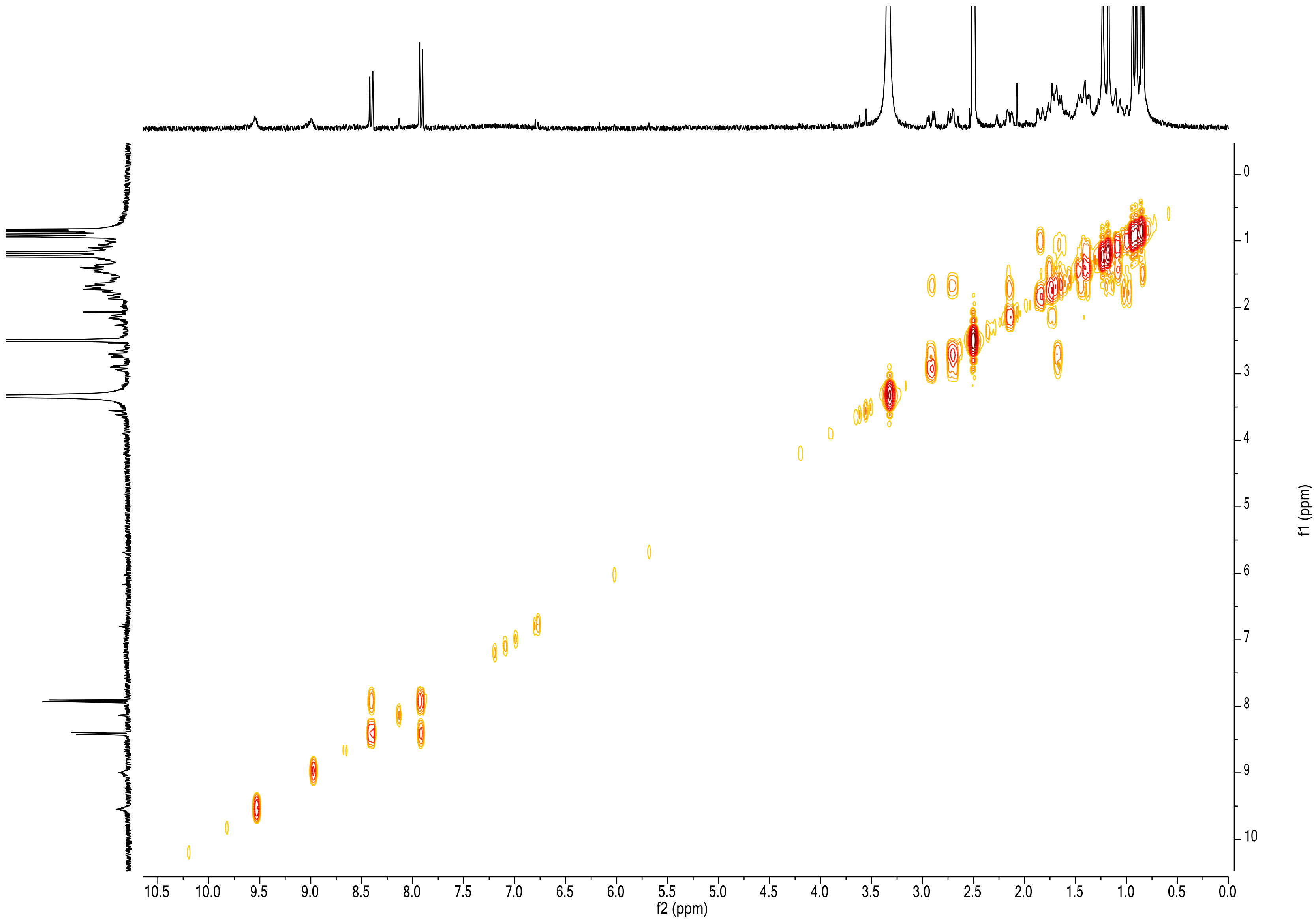

Figure S4. gHSQC Spectra (500 MHz) saccharoquinoline (**1**) in DMSO-d_6

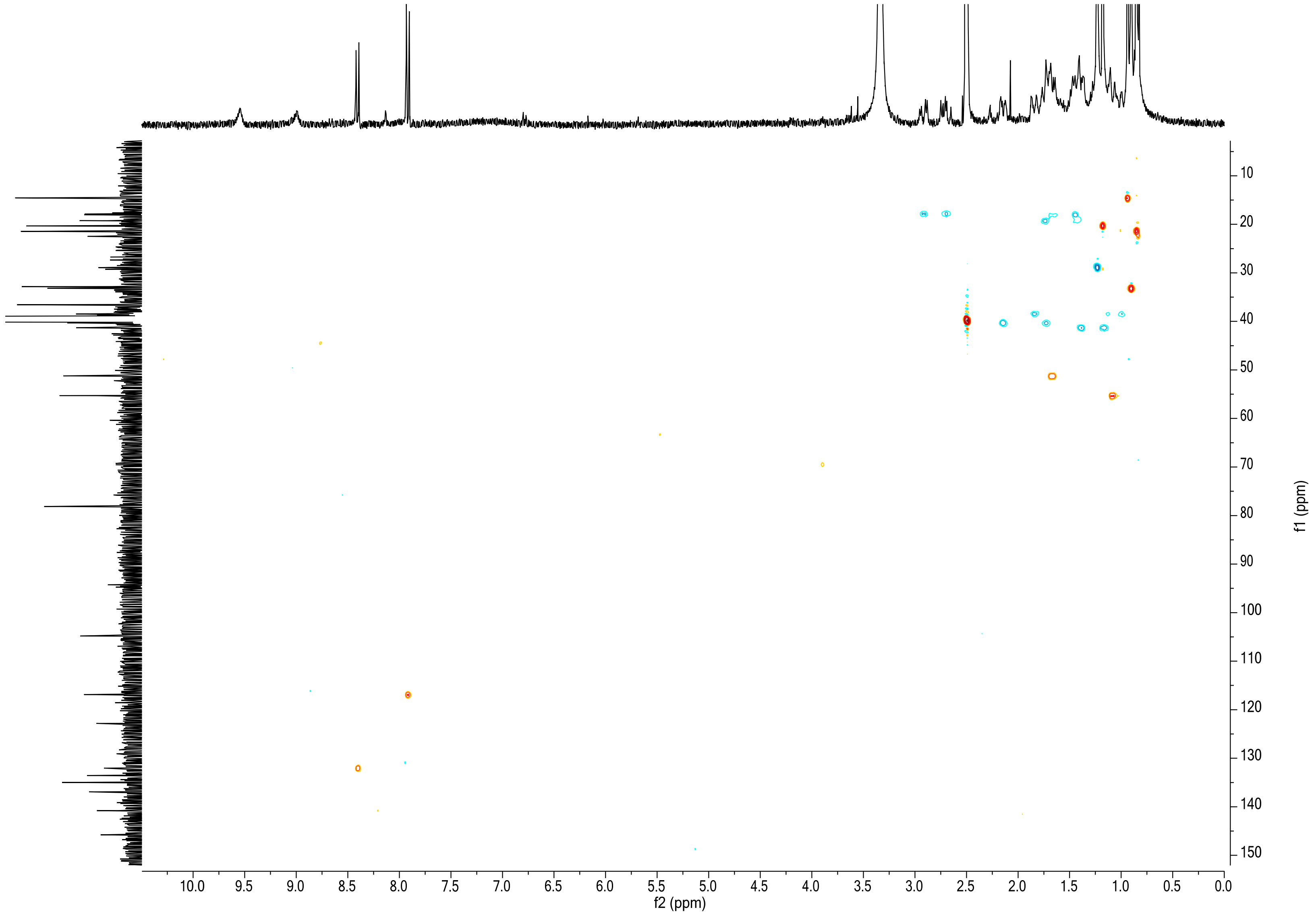

Figure S5. gHMBC Spectra (500 MHz) of saccharoquinoline (**1**) in DMSO-d_6

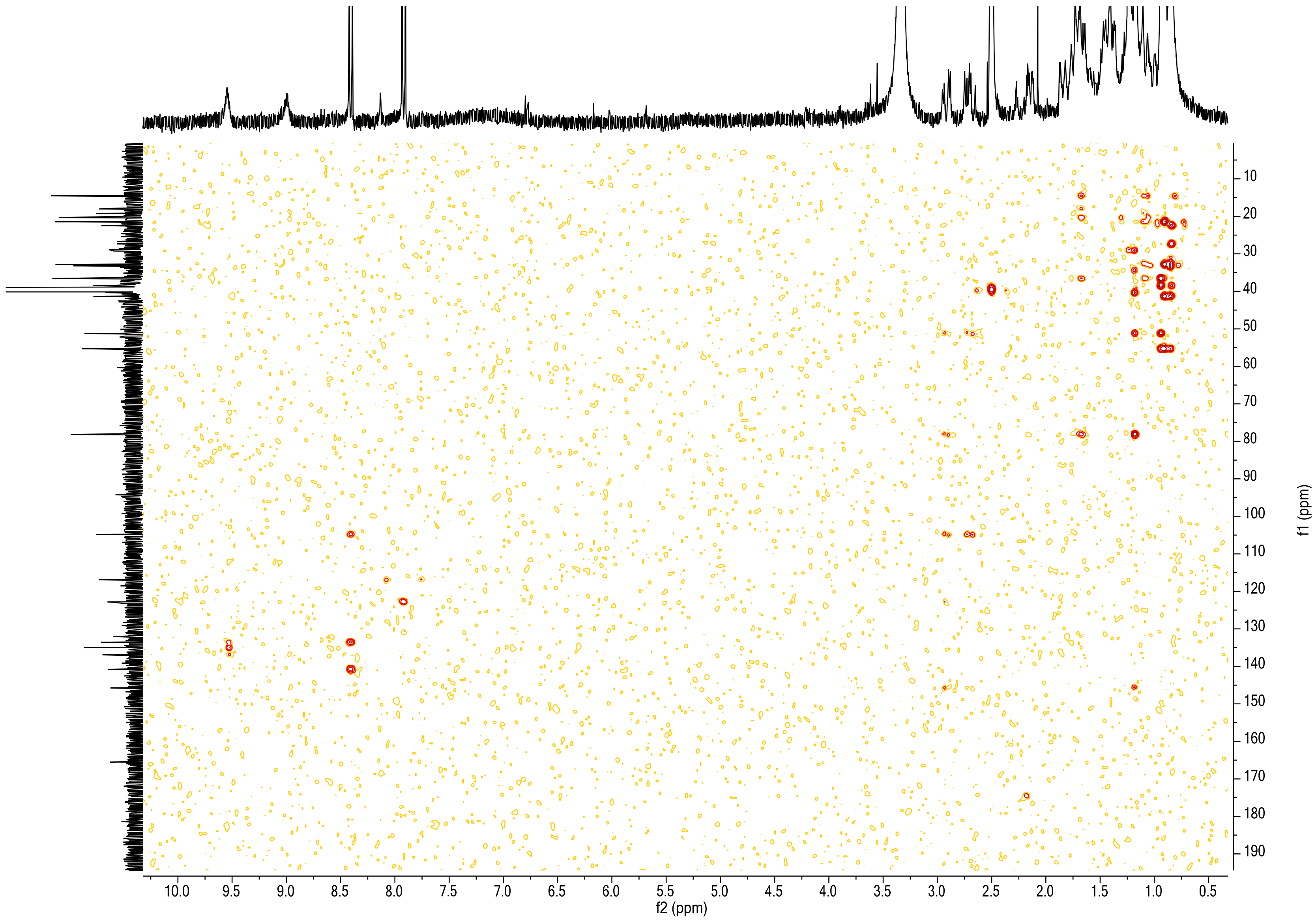

Figure S6. NOESY Spectra (500 MHz) of saccharoquinoline (**1**) in DMSO-d_6

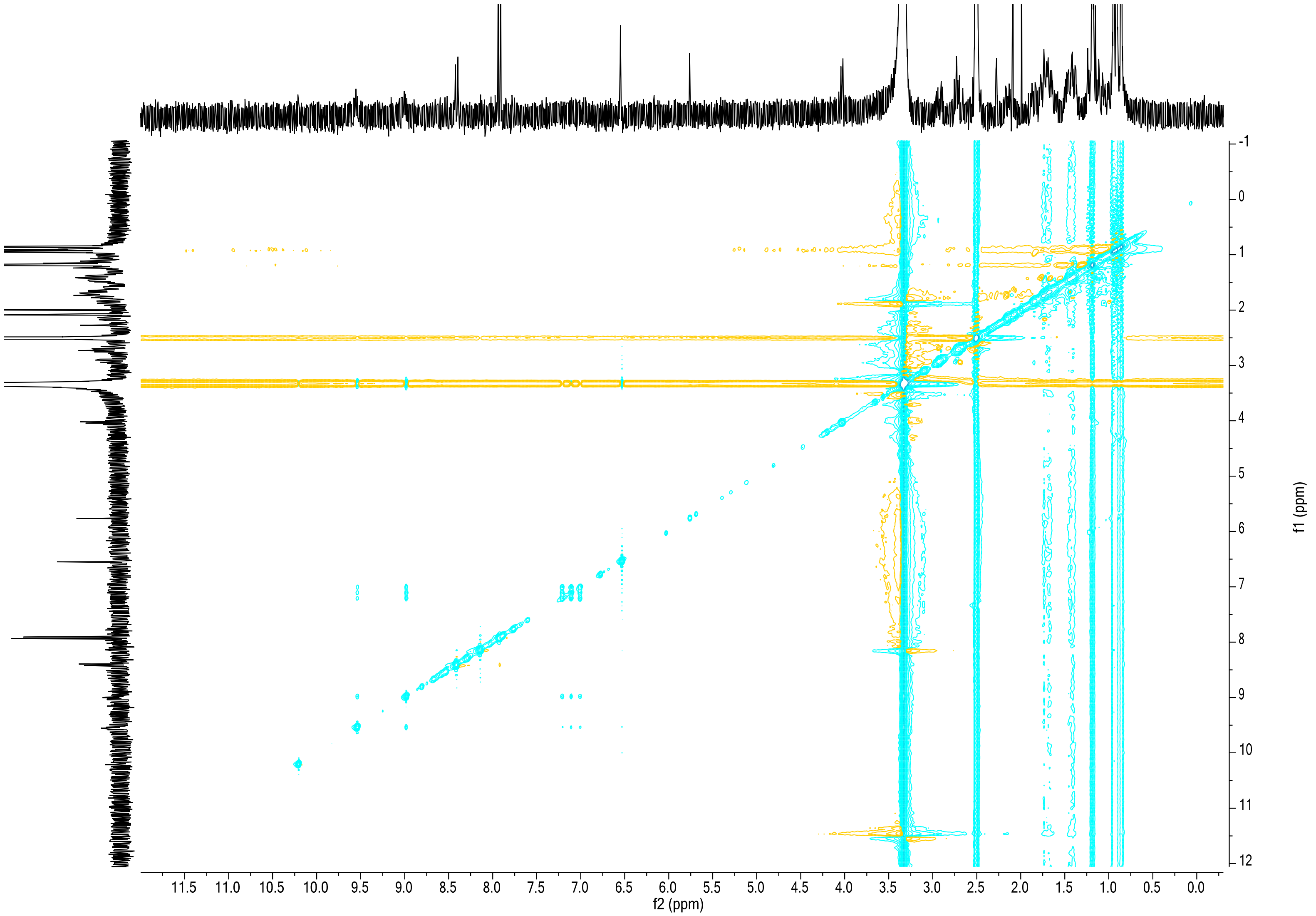

Supplement: Supplementary file 1 [file marinedrugs-17-00098-s001.pdf]
